# Looking Back, Looking Forward: Progress and Prospect for Spatial Demography

**DOI:** 10.1007/s40980-021-00084-9

**Published:** 2021-05-20

**Authors:** Stephen A. Matthews, Laura Stiberman, James Raymer, Tse-Chuan Yang, Ezra Gayawan, Sayambhu Saita, Sai Thein Than Tun, Daniel M. Parker, Deborah Balk, Stefan Leyk, Mark Montgomery, Katherine J. Curtis, David W. S. Wong

**Affiliations:** 1 Department of Sociology and Criminology, Department of Anthropology, Pennsylvania State University, University Park, PA 168702-6211 USA; 2 Department of Sociology and Criminology, Pennsylvania State University, University Park, PA 168702-6211 USA; 3 School of Demography, Australian National University, Canberra, ACT 2600 Australia; 4 Department of Sociology, Arts and Sciences 351, University at Albany, 1400 Washington Avenue, Albany, NY 12222 USA; 5 Department of Statistics, Federal University of Technology, Akure, Nigeria; 6 Faculty of Public Health, Thammasat University, Lampang, Thailand; 7 Mahidol-Oxford Tropical Medicine Research Unit, Faculty of Tropical Medicine, Mahidol University, Salaya, Thailand; 8 Centre for Tropical Medicine and Global Health, Nuffield Department of Medicine, University of Oxford, Oxford, UK; 9 Department of Population Health and Disease Prevention, Department of Epidemiology and Biostatistics, University of California, Irvine, USA; 10 CUNY Institute for Demographic Research, Marxe School of International and Public Affairs, Baruch College, City University of New York, New York, USA; 11 Department of Geography, University of Colorado, Boulder, USA; 12 Institute of Behavioral Science, University of Colorado Boulder, Boulder, USA; 13 Department of Economics, Stony Brook University, and Population Council, New York, NY USA; 14 Community and Environmental Sociology, University of Wisconsin-Madison, 316B Agricultural Hall, 1450 Linden Drive, Madison, WI 53706 USA; 15 Geography and Geoinformation Science Department, George Mason University, 4400 University Drive, MS 6C3, Fairfax, VA 22030-4444 USA

## Editorial Introduction

In 2011 a specialist meeting on the “*Future Directions in Spatial Demography*” was held in Santa Barbara, California (Matthews, Goodchild, & Janelle, 2012).^1^ This specialist meeting was the capstone to a multi-year National Institutes of Health training grant that had supported workshops in advanced spatial analysis methods increasing used by population scientists.^2^ Early-career scholars who had participated in the training workshops and senior demographers and geographers drawn from across the United States participated in the specialist meeting.^3^ The application process to attend the 2011 meeting, required that each of the forty-one attendees submit a statement that reviewed challenges and identified new directions for spatial demography, including gaps in current knowledge regarding innovations in geospatial data, spatial statistical methods, and the integration of data and models to enhance the science of spatial demography in population and health research. Reading again some of the ruminations of these scholars is an interesting exercise in its own right. The level of optimism back in 2011 was high, and especially regarding anticipated changes in computational capacity, leveraging big data (including volunteered geographic information), developments in data systems (including new data high resolution data products and online resources such as multi-scale map interfaces and dashboards), and in methods such as time–space models, agent-based models, microsimulation, and small-area estimation. There were also several challenges identified including, but not limited to, study designs, data integration, data validation, confidentiality, non-representative data, historic data, definitions of place, residential selection and mobility as well as two overarching challenges related to the role and contribution of spatial demographers in interdisciplinary population and health research, and many, many comments on training issues. Substantively the attendees research focused on all forms of interaction between people and place (and the reciprocal relations between the people in social, built, and physical environment contexts) covering the gamut of demographic processes from reproductive health to mortality, though with perhaps an overrepresentation of researchers in areas related to population and environment research, racial and residential segregation, and migration.

A decade later, it is useful to *look back* on this meeting and reflect on recent past achievements in the field of spatial demography^4^ and to *look forward* to emergent and new developments and the challenges we might expect in the next decade through 2031. We recognize that researchers can have different perspectives, thoughts, and opinions on “progress” and the future “prospect” for field of spatial demography. To help identify past achievements, research priorities and gaps, and emerging themes we reached out to several members of the *Spatial Demography*’s editorial board who attended the 2011 meeting as well as to other members of the journal’s editorial board who did not.^5^ We wrote to each contributor asking them to write a short commentary on their thoughts and insights, with requests to identify some of the publications, software, methodological developments, and data products that may influence the field going forward.

Included here are seven short commentaries. While there are differences in emphasis across the commentaries, all, in one way or another, look for greater integration of theory, concepts, and method, and more attention on data, sampling, and measurement issues. They celebrate and anticipate new software developments (including ease of access) and they echo the ongoing concern over training in spatial thinking in general and not just the narrower provisioning of training in the handling of complex spatial data and application of sophisticated spatial methods. Connecting all commentaries are theoretical and modeling based discussions of spatial interaction and spatial networks, spatial dependence, spatial heterogeneity, nonstationarity, uncertainty, place, and scale … and time or dynamics … all as they apply to contemporary and anticipated demographic questions. These shared connections between commentaries are important as even ubiquitous concerns regarding “training” can include similar but different meanings or priorities. For example, on the one hand, opportunities for training in spatial methods are often limited or centralized, especially in African and SE Asian contexts (see commentaries by Gayawan and by Saita, Tun and Parker). On the other hand, training deficiencies for demographers also can have implications more generally around their participation in big ideas/big challenge research topics and for how the field as a whole engages in critical studies of people in places and time. Indeed, while substantive demographic topics weave through all commentaries it is worth highlighting the commentaries by Balk, Leyk, and Montgomery, and, by Curtis. The former express their dismay at the lack of engagement by demographers—relative to other social scientists—in the urban systems research, especially in middle and low-income countries and the latter calls for a critical evaluation of our dominant frames, and the need for a more critical reorientation of the field of spatial demography.

Collectively, these commentaries are generally optimistic about the collective achievements in the past decade and they all identify opportunities for growth. There is also a strong sense of there still being much to be done to raise the visibility of spatially informed demographic research and thereby demonstrate the relevance of spatial demography. As you will read below, several commentaries are calls to action. Are spatial demographers up for it?

## Future of Spatial Demography

*James Raymer*

In this short commentary, three aspects of spatial demography are discussed: its unique distinction and importance in relation to other fields of spatial enquiry, current challenges, and future directions. The ideas expressed below reflect a recent paper I co-authored with Frans Willekens and Andrei Rogers on this topic (Raymer et al., 2019).

### What is Spatial Demography and Why Do We Need It?

Early developments in spatial demography connected populations through the processes of origin–destination-specific migration and allowed populations to simultaneously evolve (Rogers, 1968, 1975; Rogers & Willekens, 1976; Wilson, 1974; Wilson & Rees, 1974a, 1974b, 1975). This analytical framework greatly enhanced our understanding of how populations interact across space and change over time, and is one that should be revisited now that we have much better data, computing power, and models for capturing spatial dependency.

Fundamentally, spatial demography is the study of how populations and their compositional structures change and interact across space. At the individual level, the timing and location of demographic outcomes are linked to transitions in the individual life course (Willekens, 2014). For instance, the loss of a job or completion of education or training in one place may trigger migration to another place or, conversely, a migration may result in family separation or unemployment in the destination location. While related, spatial demography can be distinguished from both population geography and demography (Raymer et al. 2019). Population geography focuses on locational aspects of places and their influence on populations. Demography focuses on the mechanisms of population change but traditionally treats each population independently from other populations distributed in space.

A spatial demographic perspective is critical for understanding many complex social processes that are occurring. For example, populations living in cities and rural areas are struggling to adapt to major demographic developments occurring worldwide: longer and healthier lives, low and postponed reproduction, large and diverse immigration streams, increasing attractiveness of state capital cities as places to live and work, and declining propensities to migrate within developed countries (Champion et al. 2018). However, despite these trends, people are not working for considerably more years and are worried about who will support their pensions as they age. Young adults, and especially women, are struggling to balance building successful careers and raising children. Governments and individuals are concerned about the pressures of large numbers of migrants on social cohesion and urban infrastructure, including traffic and housing costs. On the other hand, regional towns are worried about their future prospects of finding labor and keeping their populations from disappearing. And, employers are having to seek labor from around the world for both low-skilled and highly specialized jobs.

To address the above issues and the interplay between demographic and societal change, spatial demographic research is needed so that we may develop theories of spatial population change, produce more robust local population forecasts, and to develop sound policies for assisting areas that are experiencing change. Key to this is the notion that populations are intricately connected to each other across space and time (Bell, 2015), and that movements between subpopulations affect both populations simultaneously, but in profoundly different ways. Spatial demography offers such possibilities.

### What are the Current Challenges?

Data availability represents a key challenge for spatial demography, especially when the interest is in understanding a large number of interacting populations and how they change over time. When disaggregated by age and sex, often the population counts and demographic events become small, which increases the effects of both random behavior and the chances of disclosure (identification). Many statistical offices are not allowed to release detailed spatial data for reasons of data confidentiality, which limits analyses. Furthermore, as shown in my recent paper published in *Spatial Demography* (Raymer et al. 2020), data on age-specific rates of fertility, mortality and migration (interregional and international) for subnational populations may not be available or measured appropriately for demographic accounting; they come from different sources (censuses, vital registers, administrative sources). Indirect estimation methods, such as combining data from multiple data sources, may be used to overcome some of these problems. They may also be used to estimate detailed components of demographic change for small areas or subpopulations (for example, by citizenship status or skill level).

### What Should We Focus on in the Future?

We need to further advance our models for spatial demography. Ideally, this would involve integrating recent developments in spatial dependency modelling into the multiregional demography framework (see, e.g., Rogers, 1995, 2020). While fixed-rate multiregional models have been useful for understanding the mechanisms of interacting subpopulations and the implications of particular rates within a system, they are often considered unrealistic or impractical for planning. Thus, we also require flexible models that account for uncertainty. This is a challenging issue but an important one, especially when estimating the demographic components of population change for small population areas (Bryant & Zhang, 2019; Swanson & Tayman, 2012, p. 235). Accounting for the large number of correlations present in the data can make spatial demographic models complex and difficult to estimate. The correlations include those across ages, cohorts, over time and space. Moreover, with migration, there are often correlations in the counter flows—that is, migration from *i* to *j* is related to migration from *j* to *i*.

In conclusion, I believe we need invest in research that brings together spatial thinking and demographic thinking. Too often we analyze the spatial patterns but not the underlying mechanisms of spatial change. This needs to change for us to truly understand spatial demographic processes and how they evolve over time.

## Advances in Global and Local Spatial Methods during the Past Decade and Future Needs

*Tse-Chuan Yang*

Since the specialist meeting in 2011 several developments in spatial econometrics and geographically weighted regression (GWR) have occurred. However, challenges remain for spatial demographers. In this commentary, I first discuss the advances in both global and local spatial statistical methods and then elaborate on areas I hope will improve in the decade ahead.

A global spatial analysis perspective aims to tackle spatial dependency embedded in ecological data by including a spatial lag or spatial error term in a model. Spatial econometrics models are, arguably, the most popular methods (LeSage & Pace, 2009) but their heavy focus on a continuous dependent variable is a major shortcoming. While the Bayesian modeling approaches and software programs (e.g., WinBUGS) allow discrete dependent variables (Lunn, 2000), the analysis is often time-consuming and not compatible with other statistical software. Nonetheless, the development of integrated nested Laplace approximations (INLA) (Rue et al. 2009) in the past decade not only improves computational efficiency of Bayesian modeling but also facilitates the applications of spatial econometrics (i.e., spatial autoregressive model) to non-Gaussian dependent variables (e.g., Poisson and Negative Binomial distributions). The R-INLA package (Martins, 2013; Rue, 2017) is well integrated with other spatial modeling libraries in R (R Core Team, 2014) and promotes generalized spatial econometrics modeling with an emphasis on random and spatially structured errors across space (Krainski, 2018). As many health or population outcomes do not follow a Gaussian distribution, such as infant deaths or confirmed coronavirus disease (COVID) counts, empowering spatial demographers to specify a model that fits the distribution of the dependent variable should minimize the potential bias in parameter estimation and statistical inference.

Another advance in spatial econometrics is the examination of the belief that spatial regression results are sensitive to the choice of spatial weight matrix (W). Using simulations, LeSage and Pace (2014) argue that this common belief is a myth, for two reasons. First, researchers are reluctant to attribute the changes in spatial regression results to their model misspecification and the choice of W becomes an easy target. The second reasons is the misinterpretation of spatial econometrics models with a spatial lag term (e.g., spatial lag or spatial Durbin models). Most research using these models has interpreted the coefficient estimates as if they are partial derivatives reflecting the net impacts of other independent variables on the dependent variable. However, this interpretation is incorrect (LeSage & Pace, 2009) and only recently has this issue been discussed in empirical papers (Mussa et al. 2017; Yang et al. 2015). LeSage and Pace’s work does not provide a definitive conclusion but it serves as an important step in helping spatial demographers to carefully think about the meaning of W.

A local spatial analysis perspective aims to investigate spatial heterogeneity, which refers to the unequal relationship between an independent and a dependent variable across space (Fotheringham et al. 2003). GWR has served this aim, despite criticisms about multiple testing and multicollinearity among local estimates (Griffith, 2008; Wheeler & Páez, 2010). Some advances in GWR are notable. First, semiparametric GWR allows both global and local independent variables to co-exist in a model (Nakaya, 2015). And, Multiscale GWR (MGWR) removes the restriction that all independent variables must share the same bandwidth (Fotheringham et al. 2017), which offers the flexibility to examine if the impact of one independent variable on the dependent variable is more localized than that of another independent variable. Second, quantile regression has been integrated into the GWR framework (Chen et al. 2012), which is known as the geographically weighted quantile regression (GWQR), and the bootstrap approach to statistical inference minimizes the concerns about multiple testing and statistical inferences (Chen et al. 2020). GWQR is particularly relevant to demographic and health research because the mean value of a distribution may not be of researchers’ interest. For example, with GWQR, obesity researchers can answer the question of how an independent variable is associated with body mass index (BMI) ranging between 25 and 30 (i.e., overweight), rather than the mean value, across the study region.

Although both global and local spatial statistical methods have experienced significant improvement since 2011, several challenges in spatial demography remain. First, and foremost, methodological advancements seem to outpace theoretical developments, specifically the lack of theory from an ecological perspective (Howell, Porter, & Matthews, 2016). Most theories related to demographic and health outcomes are based on individual perspectives and directly applying these theories to an ecological analysis is problematic (i.e., the ecological fallacy). It becomes critical for spatial demographers (and more broadly social scientists) to develop spatial theories based on the ecological data. Some actions may help overcome this challenge, such as encouraging the publications of descriptive findings and engaging in a discourse on how collective individual behaviors/relationships could be reasonably translated to ecological associations. Second, compared to a decade ago, longitudinal ecological data have become more readily available. However, relatively little effort has been made to improve the analysis of spatial panel (or spatiotemporal) ecological data (cf. Millo & Piras, 2012). While many sociodemographic variables may not change rapidly from one year to another, bringing the temporal dimension into spatial analysis should offer a better understanding of spatiotemporal dynamics, which goes beyond the descriptive trends across space and time, respectively.

Third, as discussed previously, the generalized spatial econometrics models are mainly for spatially structured errors, there is a need to develop software programs (and methods) that can implement the generalized spatial econometrics models with a spatial lag term under the Bayesian statistics framework, such as spatial Durbin model for discrete variables. Should this gap be filled, spatial demographers will have an extensive toolbox for global spatial analysis. Last, but not the least, spatial demographers should consider how to meaningfully define W, instead of using the conventional geographical adjacency or distance. For example, in light of the ongoing COVID pandemic, the flows of essential workers among areas (e.g., from their residential to working areas) should provide more information about how the disease transmits across space than the conventional first-order Queen or nearest-K neighbors. More is needed from spatial demographers to justify or even theorize on the use of W.

Spatial demography has benefitted from developments in spatial statistical methods in the past decade. Should the four areas or gaps discussed above be filled in the next ten years, spatial demographers will create a stronger connection between theory and method and be in a better position to advocate the importance of space and time in the field.

## The Future of Spatial Demography: Advances in Statistical Models that Support Spatial Demography Inquiry

*Ezra Gayawan*

Interests in the field of spatial demography has grown rapidly, largely due to the availability of spatial and spatiotemporal data, advances in methodology, and in computational tools. Researchers in a wide range of fields increasingly work with georeferenced data and there are now a growing number of applications examining spatial processes in fertility, mortality, migration, health, and their determinants in developed and developing countries. Regional disparities in developmental programs across developed and developing nations have also been of concern to analysts interested in spatial modeling. This is because when developmental measures are placed on maps, they reveal opportunities for identifying location-specific intervention programs, making them appealing. In Africa, the use of spatial methods in demographic and health studies has started to take root.

Most of the statistical methods underlying spatial models are extensions of the familiar statistical techniques including regression and time series models. Except in some cases where the models are allowing for the classical inferential methods, the estimation procedures are often based on Bayesian techniques; due to advances in speed and algorithm development particularly the further development of Markov chain Monte Carlo (MCMC). The MCMC method allows for flexibility in fitting hierarchical models, thus, giving room for different approaches of conceptualizing spatial patterns and processes. Further, most of these techniques are implemented in code-oriented software such as R. Some demographers and social science researchers with little background on statistical and computational methods tend to shy away from these methods (rather than confront their fears). However, there are an extensive catalog of useful texts with practical examples that allow interested researchers to link statistical theories to data problems especially those pertaining to spatial modeling. Most texts come with R-code that can easily be modified or serve as building blocks for modeling real life spatial data, thus aiding our understanding of phenomena of interest. Books I have found to be extremely useful in this regard include Blangiardo and Cameletti (2015), Fahrmeir et al. (2013) and Moraga (2020).

African researchers’ involvement in spatial demography and disease mapping has seen the publications of several useful contributions. In particular, the publication, in 2014, of the edited book, “Advanced techniques for modelling maternal and child health in Africa” (Kandala & Ghilagaber, 2014) combines both theoretical methodologies and empirical applications of hierarchical spatial models to demographic and health data from several African countries, covering a range of models from both classical and Bayesian techniques. Several chapters discuss model formulation using geoadditive predictors, which embrace regression models such as generalized additive models (GAM), generalized additive mixed models (GAMM) and stepwise regression model. The majority of the authors reside and work in Africa and have continued to advance the methods presented in the book through their teaching and research. The book itself has continued to serve as a reference point for spatial modeling by African scholars.

My journey into spatial demography was motivated by Fahrmier and his colleagues, who made extensive contributions to statistical methods generally described as “structured additive regression models (STAR)” (Fahrmeir et al. 2004; Hennerfeind et al. 2006; Kneib & Fahrmeir, 2006). These methods extend the classical regression model by offering a comprehensive approach that enables the simultaneous modeling of predictor variables of different types including nonlinear effects of metrical covariates, spatial correlation, and heterogeneity, and the linear fixed effects of categorical variables while the response variable of interest can take on one of several distributions especially those commonly encountered in demography and health studies, thus, encompassing different kinds of regression models. The initial method development has seen applications in estimating the levels of different demographic and health phenomenon of interests. However, recent extensions allow the estimations of more interesting spatial attributes of the data such as the variances, standard deviations or other parameters of interest as seen in some recent applications (Gayawan, Fasusi, and Bandyopadhyay, 2020; Somo-Aina & Gayawan, 2019). Though, as with other methods, the estimation procedures are based on fully Bayesian approaches. There are textbooks aimed at readers with average statistics backgrounds to understand these concepts and their applications (see Fahrmeir et al., 2013; Fahrmeir & Kneib, 2011 and the reference list). These authors have made available data and code to illustrate their methods and this should enhance knowledge dissemination among demographers seeking to apply regression-based models to spatial data. Though there are R packages such as R2BayesX and BayesX that enable easy implementations of STAR models in R, the software BayesX – Bayesian Inference in Structured Additive Regression Models (Belitz et al., 2015) freely available at www.bayesx.org, not only implements the methods but also provides easy-to-understand examples in its tutorial manual.

The approximate Bayesian inference for latent Gaussian models, a subset of the STAR model as implemented through the integrated nested Laplace approximation (INLA), a deterministic algorithm proposed by Rue et al. (2009) is another interesting statistical methodology that may appeal to demographers interested in spatial applications. A useful feature of this method is that it lessens computation burden especially when the estimation involves models with high spatial and temporal resolution. Unlike the Bayesian inference through MCMC that sometimes leads to several days of computing time, the INLA approach, implementable through the R package R-INLA (www.r-inla.org) provides accurate and fast results. The method is exceptionally suitable for both areal and point reference data. The later applications arise when observations are measured at finite set of specific points in a given region and the interest is to generate a continuous spatial field and also to predict values at unobserved locations or in identifying areas where the risks of exceeding potentially harmful threshold is higher.

With the growing advancement in methodological and computational tools coupled with the increasing georeferenced data, it behooves one to note that the field of spatial demography is continually evolving. Its future will be written by those who take early advantage of available resources and get involved. Rigorous training in the form of workshops and short courses seldom organized for African researchers are now needed than ever before. These would supplement the rare in-depth demography training offered by the few institutions with the in-house capacity, though these usually do not included adequate syllabi on spatial analytical techniques (Matthews, 2016). There are many evolving areas of future applications of spatial demography that could help in monitoring and evaluation of developmental programs particularly the Sustainable Development Goals (SDGs) at more localized levels as this would provide evidence for multi-sectorial policies.

## The Past, Present, and Future of Spatial Demography in Southeast Asia

*Sayambhu Saita*, *Sai Thein Than Tun*, *Daniel M. Parker*

Demographers have been considering elements of geography since the onset of demographic research (Voss, 2007). However, formal training in spatial analyses in demography has expanded in recent years; likely a result of newly available data, computational and statistical capabilities, and general growing interest among demographers (Matthews & Parker, 2013). In this short commentary we briefly explore the history of demography in Southeast (SE) Asia. We aim to be inclusive of the region, but our experiences and knowledge have largely been centered on mainland SE Asia (including Myanmar, Thailand, Lao People’s Democratic Republic, Cambodia, and Vietnam) and so we admittedly draw most heavily on these places in our examples and discussions. We close by discussing the current state of data collection and analytic training that is available in the region. We argue for the collection and distribution of better demographic data as well as for equity in the training of spatial demographic analytic approaches.

### Demography and Spatial Demography in SE Asia

SE Asia is home to diverse populations, geographies, cultures, and environments and has experienced immense change over the last century. Demography, including collection and use of geographically referenced population data, has a long history in SE Asia. The bulk of this demographic work has historically been for practical purposes and with an “applied” emphasis. The ancient kingdoms of the region kept census data and occasionally sent reconnaissance teams to report on populations in less well-documented regions (frequently in buffer zones between kingdoms) (Wilson & Hanks, 1985). Western experts in demography and statistics came with imperialism and colonialism in much of SE Asia.

In 1950–1955 total fertility for SE Asia was approximately 5.93, with Viet Nam having the lowest Total Fertility Rate (TFR) (5.4) and the Philippines having the highest (7.4). Today the situation has changed drastically, with the region having an estimated TFR of 2.22, the lowest in Singapore (1.2) and highest in Timor-Leste (4.1). The TFR decline has been heterogeneous across the region (World Population Prospects, 2019). Singapore experienced the decrease in TFR quicker than other nations, followed by Thailand and Malaysia while Lao People’s Democratic Republic (PDR) and Cambodia experienced a lag in the fertility transition. Extreme subnational heterogeneity (especially between rural and urban areas) also exists in fertility.

Declining fertility and increasing life expectancy have led to an overarching change in the population structure, and to major changes in leading causes of disease, delayed marriage, changes in frequency of cohabitation, postponement of parenthood, high divorce rates, and the size of the working age population (Prasartkul et al., 2019). For example, young skilled workers have been considered to be an essential component of regional plans for a rapid economic catch-up process over the last three decades. However, with an aging population it becomes increasingly necessary to depend on labor from high fertility areas; including nations that have not yet fully transitioned or subnational areas with continued high fertility (i.e. rural areas). Migration is therefore a major spatial demographic attribute of the Southeast Asia region (Rigg, 2013), with migrants from nations with lower economic opportunities moving toward wealthier nations, and those from poorer rural areas moving into urban areas.

All of these standard demographic processes (birth, death, migration) exhibit spatial and temporal heterogeneity, as well as spatial dependencies, at multiple spatial scales: regionally, nationally, and sub-nationally. Spatial demography is therefore crucial for understanding historical and current demographic patterns, for estimating demographic indicators and phenomena in poorly sampled subregions, and for peering into the future of the SE Asian demographic landscape.

Two major spatial demographic needs should be addressed for SE Asia: data and capacity.

### Collection of Demographic Data

Thailand has one of the longest ongoing population census programs in the region. In 1905 a population census was undertaken, during the reign of King Rama V and under the administration of the Ministry of the Interior, though it covered only 12 precincts out of a total of 17 administrative precincts. Some colonial authorities carried out population censuses, though quality was frequently of questionable nature (French Indo-China, 1945; Hirschman & Bonaparte, n.d.). After independence, most nations established their own offices of statistics with one goal being to undertake and maintain population censuses. The United Nations has been instrumental in aiding the collection, analysis, and dissemination of census data. However, regional and civil conflict, lack of resources, and difficulties in reaching some populations have meant that gaps remain in the data. In some cases, demographic health surveys (DHSs) (U.S. Agency for International Development n.d.) or other targeted surveys have been helpful in filling some data gaps (Corsi et al., 2012; Mutunga et al., 2020). DHSs have been conducted in Cambodia, Indonesia, Lao PDR, Myanmar, Philippines, Thailand, Timor-Leste, and Vietnam. DHS programs tend to be limited in their spatial and temporal coverage, but often offer data that are collected at high spatial resolution that is useful for micro-level spatial demographic analyses.

Increasingly, mathematical modeling and geostatistical approaches have been used to estimate population distributions across the SE Asia landscape, including in areas where empirical data are scarce. Such estimates are extremely valuable, but are not a substitute for high quality census data. Some estimated population distributions are at granular scale (Gaughan et al. 2013) but have several limitations. For example, where these datasets fill in important gaps in the data, validation of these estimates in remote areas and conflict zones is frequently not possible. Working with individuals from these areas can help to address some of these gaps, perhaps especially with regard to data validation. As political and civil stability can be quite fluid, working with people from affected areas can also be useful for planning on-the-ground data collection when it is feasible.

This importance of high-quality demographic data with granular spatial attributes cannot be overstated. There are no adequate demographic or epidemiological indicators in the absence of proper denominators. Furthermore, given the age- and gender-specific risks that are characteristic of many health outcomes, more detailed data are necessary (Leyk et al. 2019). Recent gridded data (from WorldPop, etc.) do include age and gender breakdowns (Tatem, 2017). However, even more disaggregated data are needed for application. Nation, province, and district level are far from sufficient for programs (family planning, public health, or otherwise) that ostensibly operate at more disaggregated units. Community-based clinics, for example, need population estimates (including age and gender) at the community scale.

While high quality population estimates exist for SE Asia, they should not be the default approach to demographic data. SE Asia has drastically changed in recent years and it is now possible to collect empirical data in places that were not previously reachable (Parker et al., 2017). These data also need to be consistently updated, ground-truthed, and need to be usable at different geographic aggregations. Population registries with data that are continuously updated, similar to those in the Nordic countries (United Nations Economic Commission for Europe, 2007), could be a goal for which SE Asian nations aim.

### Capacity Building for Spatial Demography in SE Asia

Examples of sophisticated spatial demographic work in SE Asia are numerous; too numerous to be adequately presented in a short comment. Included in this body of literature are research programs that were and continue to be cutting edge, including: detailed studies of the interactions between population settlements and environment (Walsh et al., 2005, Entwisle et al., 2008, Kermel-Torrès, 2020, Schönweger, 2012), sophisticated analyses of migration movements and settlements (Shao et al., 2008), novel approaches to measuring access to family planning services (Entwisle et al., 1997, Rittirong et al., 2013), detailed work on changing fertility patterns in places where data are scarce (Schuster et al., 2019), and the generation of gridded population estimates across the region (Gaughan et al., 2013.) This list is not exhaustive, but rather is meant to illustrate the wealth of spatial demographic work that has been done in this region.

Nevertheless, shortcomings remain. While high resolution data are available for some nations (e.g. village level for Lao PDR), the data are much more course in other, neighboring nations (e.g. only region or state level for Myanmar). The factors that drive the heterogeneity in the granularity and even quality of data across the SE Asian landscape are numerous, fluid, and complex. Economic capacity is a major driver, with limited funds limiting laborious data collection, cleaning, and management. Conflict and difficulties in accessing some locations also drives these problems. Concerns with regard to confidentiality and other political sensitivities may also lead to the diminished availability of demographic data for small geographic units.

Furthermore, much of the sophisticated spatial demographic research in SE Asia has been led by researchers who are neither from, nor based in, SE Asia (though typically at least including SE Asian authors). This issue is echoed in other fields where international work is common, and has recently gained much attention in Global Health (Iyer, 2018; Sheikh et al., 2017). While this issue is often framed as one that exists between high income nations and low- or middle-income nations, disparities in spatial demographic research capacity also exist between and within SE Asia nations. As an example, Thailand has a well-established system to record demographic data and celebrated the 100th anniversary of the official census in 2010 (National Statistical Office: Thailand, n.d.) while Myanmar restarted its census in 2014 after 30 years of hiatus (Population Censuses in Myanmar, n.d.). Several population research and training centers exist in the region, including: the Institute for Population and Social Research, Mahidol University, Thailand; the College of Population Studies, Chulalongkorn University, Thailand; the Singapore Population Health Studies, Saw Swee Hock School of Public Health, Singapore; Population Studies Unit, University of Malaya, Malaysia; Biostatistics and Population Studies, University of Indonesia; and the Population Institute at University of the Philippines. These centers tend to be located in relatively wealthy nations in SE Asia. Even within nations that have high capacity for collecting and analyzing spatial demographic data, research programs tend to be centered in major metropolitan areas, with occasional research programs that focus on rural populations but often fail to include rural people as researchers.

Likewise, training programs, including workshops that focus on sophisticated spatial analytic approaches, do exist and occur in the region. They are often located in major institutes in urban centers, with the bulk of trainees also coming from large, prestigious institutes and from urban centers. While high quality, open source and free analytic software are now widely available—many of these training programs continue to use proprietary and frankly expensive software systems. This practice puts trainees without sufficient financial resources, or without ties to institutions that can pay the needed fees for such software, at a major disadvantage. Whereas it once could be argued that these expensive software systems were necessary for conducting more sophisticated analyses, at this point it is more likely to be based on trainer preferences. Free software systems such as the R statistical software system (R Core Team, 2014), Quantum GIS (QGIS.org, 2021), GeoDa (Anselin et al., 2010), and CrimeStat (National Institute of Justice, 2019) (and more that we have not listed) are together capable of most sophisticated analytic approaches. Training of spatial demographers in low and middle-income nations and regions should now use these types of systems.

Ultimately, what is needed are training programs and systems that offer to train students in sophisticated analytic approaches, are accessible to all, and which use statistical and geographic information systems software platforms that do not require fees (which only exacerbate the problem in economically poor regions and subregions). We recommend an equitable approach to spatial demography in SE Asia, for a variety of practical and moral reasons. Training for the next generation of leaders in spatial demography of SE Asia should be inclusive of folks from SE Asia, and importantly from parts of SE Asia that have historically been underrepresented in the literature and field. Doing so will likely lead to stronger research output, with deeper understandings of the contexts of places, with an enhanced ability to problem solve data collection and the interpretation of complex analytic results, and for ensuring that strong data collection and analysis continues well into the future.

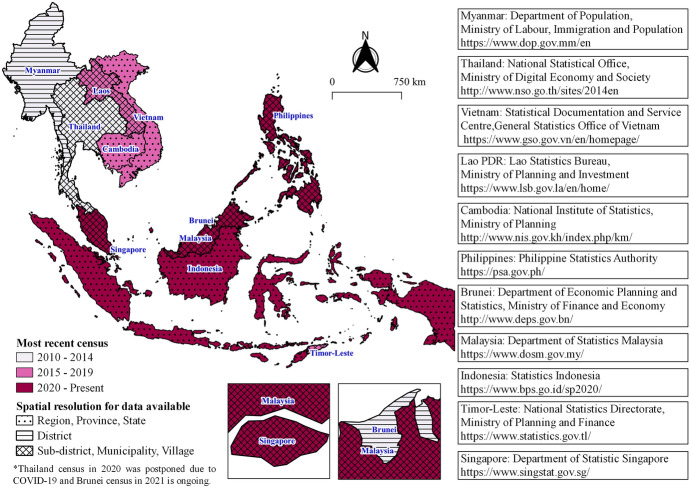



**Figure 1** Responsive organization and data available for the national censuses in SE Asia.

## Revitalizing Urban Research: What Is The Future Role of Demographers?

*Deborah Balk*, *Stefan Leyk*, *Mark Montgomery*

Over the foreseeable future, a steadily rising proportion of the world's population will be coming to live in geographically small, high-density communities—that is, in cities and towns. Urban places are not only clusters of high population density: They exhibit similar densities in physical and human capital, educational and health facilities, and the levels and units of government. If access to beneficial resources could be measured by geographic distance alone, then urban residents would enjoy a decided advantage. But especially in large cities, neighborhoods range from the comfortably advantaged to the distressingly disadvantaged; the residents of disadvantaged neighborhoods can be socially and economically excluded from resources that may be no more than a stone's throw away. Urban spaces are also crisscrossed by a great variety of social, economic, and transport networks that connect people inhabiting different neighborhoods, workplaces, and residences. The possibilities for social linkage thus depend on resources situated across an array of physical sites.

Viewed in this way, cities and towns of various sizes and forms offer multiple opportunities for the fine-grained, multi-level analyses at which demographers excel. And yet, apart from the Chicago School demographers who have mainly studied the cities of high-income countries, very little of the field's attention has been directed to urban communities elsewhere in the world. This is, frankly, inexplicable. The vast majority of the world's upcoming population growth is projected to take place in the cities and towns of low- and middle-income countries. Spatially-detailed empirical measures of the urban–rural continuum and connectivity have been flooding into the literature over the past decade, enriching the possibilities for understanding and modeling behavior across the full range of rural and urban landscapes. In poor countries, large within-urban and urban–rural differentials have already been documented in demographic behavior and outcomes, including age at marriage, fertility, use of contraception and unmet need, rates of abortion, children's education, and both infant and child health and mortality (Montgomery et al., 2003). Across all these dimensions, the situations of the urban poor more closely resemble those of rural-dwellers than the urban non-poor.

Little is yet known, to be sure, of the roles that might be played by neighborhoods and social networks in these demographic domains. The prominence of migrants and the residentially mobile in the urban scene—people whose ideas and resources are drawn from life-experiences in multiple places—adds to the analytic challenge. Perhaps as many as half of those forcibly displaced by civil strife or environmental disruption eventually make their way to slums and poor urban communities (Forced Migration Review, 2020), and from them there is doubtless much to be learned about shocks, adaptation, fragility, resilience, and mental health. From a demographer's point of view, it is hard to survey this panorama of issues and imagine a more inviting scientific prospect. Yet to date, demographers seem to have declined the invitation.

In 2015, in the introduction to a special issue in this journal echoing many of the same themes (Balk & Montgomery, 2015), we called on demographers and allied social scientists to reappraise their concepts, methods, and data in order to improve the fit between our disciplines and the emerging demographic realities (Montgomery & Balk, 2011). While some positive changes have occurred—notably in the improvement of sampling frames in major survey programs to capture slum-dwellers, and the continued commitment to providing geospatial data on both survey respondents (albeit with intentional dislocation that compromises analytical use in urban areas) and small census units—the major changes of the past decade have taken place outside demography proper. Geographers, remote-sensing experts, geophysicists, and ecologists have made great progress in improving models and data on the spatial extents of the human built-up environment and its interactions with non-human environmental processes (Leyk et al., 2020; McDonald et al., 2011; Pesaresi et al., 2016; Small, 2020; Small et al., 2018; Montgomery et al. in press). Epidemiologists have drawn on mobile-phone and social media data to trace out social networks and approximate short-term migration flows (Tatem, 2014). It is now far easier than it was a decade ago to describe within-urban environments using resources such as OpenStreetMap (e.g., Barrington-Leigh & Millard-Ball, 2020; Boeing, 2020). These new data enable the shifting boundaries of urban spaces to be delineated in far greater detail than was previously possible, and shed much-needed light on the interior organization of these spaces.

Apart from the use of mobile-phone data, less has been accomplished with regard to social and economic networks, and little progress is evident on migration and residential mobility. Clearly, much remains to be done. In our view, however, the first-order priority is to establish the spatial frame within which urban demographic behavior takes place. In an earlier commentary (Balk, 2011), we argued that the demographic study of urbanization requires that demographers and allied fields adopt spatial tools and methods as their own, beginning by integrating key geographic concepts in their thinking. While spatial approaches are becoming more widespread (Balk et al., 2018; Donaldson & Storeygard, 2016; Henderson et al., 2012), especially in studies on the interactions of population and the environment (Kugler et al., 2019; Liu & Balk, 2020), they remain far from commonplace and are mostly implemented by researchers from allied social science disciplines.

With respect to studying urbanization, not only have great strides been made outside the population sciences on the spatial delineation (and change thereof) of the built-environment (Corbane et al. 2019; Gao & O’Neill, 2020; Uhl et al., 2021), but because demographers have largely been quiet in this domain, core concepts, data resources and models are evolving with limited benefit of demographic thinking (Leyk et al., 2019). It is something of a mystery why demography—a field that prides itself on its interdisciplinarity—has engaged in so few collaborations with the geographic, environmental and data sciences.

Given the above arguments, we propose the following:

1. Demographic training simply must cover and integrate spatial concepts, methods, data, and tools.
2. Demographers should engage with place-based constructs and processes, and make room in their thinking for urbanization alongside conventional demographic concerns such as aging, fertility, children’s education, migration, and mortality.
3. Demographic data collection for the twenty-first century needs to be re-imagined: sampling frames need to consider the full urban continuum, and be capable of disaggregation to the neighborhood level, so as to systematically address inequality, segregation and vulnerability within cities.
  - Ideally, the sampling frames of the twenty-first century should also align with and thus represent the natural environments (such as watersheds or coastal zones, which are expected to be under substantial change during this century) that are disproportionately home to or influenced by city dwellers (McGranahan et al., 2007).
4. Demographic data along the urban continuum must be spatially rendered at appropriate scales, using best-practice methods to ensure consistency in their spatial and thematic properties, regardless of whether these data come from censuses, surveys, surveillance systems, or administrative records.
5. Recognizing that the study of urbanization is informed by both population and land-use perspectives, demographers working in this domain must go further than other population scientists in making spatial methods and data part of their craft and engage with the scientific communities now making tremendous progress in integrating remote sensing and population data (along with other ‘big data’).

There is some urgency to this call. Those engaged in work on the urban–rural continuum will continue to work without the full benefit of demographic insights so long as the demographic community continues to stand aloof. The potential for mutual gain is obvious: demographers would benefit from access to data more relevant to urban well-being and urban structure and from the integration of spatial urban concepts; non-demographers would benefit from a fuller understanding of demographic processes and from the rigorous application of demographic analytic techniques to a wider array of accessible data. If such engagement can finally take place, it will surely produce a much richer and deeper understanding of our collective urban future.

## Future Spatial Demography—A Critical Moment

*Katherine J Curtis*

Spatial thinking and spatial analysis play a central role in most questions principal to demography. Many a scholar has convincingly asserted the inherent and explicit spatial nature of the events and processes studied by demographers (e.g., Entwisle, 2007; Voss, 2007; Weeks, 2004). These phenomena are among the most socially relevant and, at times, highly political issues confronting populations and the particular places in which they are embedded, all around the globe. Now is clearly one of those times. Currently, we are confronting the global pandemic of COVID-19 and, not unrelatedly, the on-going pandemic of systemic racism and the unjust violence it provokes. Demographers have much to say about both. My concern centers on what we demographers have to say, the means through which we generate our contributions, and precisely to which arguments we are contributing. I am calling for a critical evaluation of our dominant frames, and a critical reorientation of our field. Spatial demographers are uniquely positioned to lead this charge.

A decade ago, as a collective of specialists we spatial demographers identified the need to integrate spatial training into basic, required demographic coursework. Without such integration, advances in spatial demography will be stymied if not marginalized, pursued by a small choir who mostly sings to itself. The final report noted that the issue of training emerged in nearly every plenary and breakout session, and highlighted the necessity of institutional level cooperation and investment for meaningful change in training (Matthews et al., 2012).

I am among those in the choir who has worked to integrate spatial thinking and spatial analysis into demography. Taking a broad view on training, my efforts range from formal semester-long courses and several-day workshops, to on-going working groups and one-off seminars, to directing or contributing to graduate master’s and doctoral committees, to publishing in field journals. In each of these pursuits, I see a need for more theoretical development in spatial thinking, and I see push back and barriers to such development. It is among these needs and the obstacles to meeting them that give rise to my broader concern about a lack of critical perspective in demography.

In my experience, at the core of the obstacles to spatial training is the diminishment of place-based questions and an instilled yet irrational fear of the ecological fallacy. As a result, our field falls short in several, connected detrimental ways. First among them is that we fail to seriously engage in critical perspectives of place-making and place-meaning. Consequently, our field perpetuates a scientific literature that pathologizes and stigmatizes places and populations systematically marginalized by oppressive forces (i.e., those of the racist, classist, gendered, and heteronormative types).

Second, and relatedly, we fail to identify appropriate and meaningful spatial units and scales. Consequently, we conflate the census tract or other arbitrary administrative units with socially or politically meaningful places. And, we do this despite continued work demonstrating the relevance of alternative units at different, related scales (e.g., Fowler et al., 2016). We generate work that emplaces populations in statistically relevant polygons or centroids that do not readily translate into social landscapes within which power relations play out.

Third, we engage in implicitly spatial work as opposed to explicitly spatial work. Using the word “spatial,” spatial units of analysis, or robust standard errors does not equate spatial thinking, spatial theorizing, or spatial analysis. This issue is more than pedantic. It is an issue of study perspective, which determines the questions asked and the answers found.

Fourth, and closely linked to the previous failing, we have not taken seriously spatial heterogeneity as a conceptual centerpiece. As a result, we perpetuate universalist assertions that mask systematic and predictable spatial differences in outcomes and their contributing forces. In doing so, we ignore diverse experiences, outcomes, and relationships.

Each of these failings amounts to a limited if not potentially harmful science. They have generated a science myopic in its understanding of the complex social world and, in turn, its capacity to address the problems confronting myriad and distinct places and their populations.

There is a way forward, and I am suggesting that it is through adopting a critical spatial demographic lens.^6^ This suggestion is neither new nor even radical, but rooted in the foundational work of scholars outside of demography. In my disciplinary home of sociology, critical race scholars have emphasized the work of W.E.B. Du Bois, which positions the color line as global, place-specific, and entrenched in unequal power relations resulting from colonialism (Itzigsohn & Brown, 2020). Importantly for spatial demographers, Du Bois’s approach emplaces people in their historical and spatial contexts.

Du Bois’ work, and that by scholars informed by him (e.g., Zuberi & Bonilla-Silva, 2008), works against the discipline’s normative treatment of Black people as problems. While not all demographers are sociologists, demographers are equally habituated in problematizing Black populations (and other populations of color) and the places they reside, even when the research is intended to address racial inequality. An example of this is the pathologizing narrative that dominates the literature addressing neighborhood effects on health. It is within Du Bois’s approach to community and urban studies that a potential pathway for spatial demographers exists. Here, guiding principles include studying local areas rather than national aggregates in order to capture heterogeneity in social environments and embedding populations within (historical) networks that make a place, its opportunities and its constraints (Itzigsohn & Brown, 2020).

Du Boisian ideas about heterogeneity and historical contexts draw parallels with the instrumental work of geographer Doreen Massey who similarly emphasizes spatial heterogeneity and temporal dynamism, but challenges the gendered systems of meaning underlying scientific practice. Massey’s work reveals how particular ways of thinking about space and place are tied up with particular social constructions of gender relations, which can be extended to race relations, class relations, and other relations derived from social relations (Massey, 1994). Drawing from Massey, we can understand spatial relations and spatial processes as social relations taking a particular geographical form (see Neely & Samura, 2011). Massey argues for the consideration of the local, specific, concrete, and descriptive, which is opposite of the universal, abstract, and generalizing science. She asserts the former approach is coded feminine while the latter is masculine, and this symbolic association gives rise to the devaluation of the former. This devaluation is among my list of our field’s failings.

Part of the way forward for spatial demography can be found in these foundational sociological and geographical perspectives. Another part of the way forward is reconciling the ongoing influence and limiting perspective of the Chicago School in spatial demography. Each of the failings I identify are linked to this school of thought and practice. I, myself, was trained under the Chicago School doctrine, yet I come from a place that cannot be adequately represented through this lens. This doctrine promotes an ahistorical, universalist perspective that excludes differences, agency, and delegitimizes alternative standpoints (Itzigsohn & Brown, 2020). What we need is a framework shift that incorporates the socio-historical forces that make and give meanings to place, reflects on appropriate spatial units and scales, is explicitly spatial in its theory and methods, and builds from a spatial heterogeneity orientation. Such a pathway might lead us to answering questions that matter for the populations and places we spatial demographers presume to understand and empower.

## Measuring the Patterns of Segregation: A Decade of Progress

*David W. S. Wong*

In “Perspective on Future Directions for Spatial Demography” written for the 2011 Specialist Meeting, I proposed future research directions in three intersecting areas in spatial demography-population geography: measuring spatial segregation, spatial pattern analysis related to scale, and the use of American Community Survey (ACS) data. This commentary provides a casual assessment on the progress made in these areas in the past decade.

Two recent reviews of segregation measurement I was involved in focus on the technical aspects in measuring segregation from a spatial perspective (Oka & Wong, 2019; Yao et al., 2019). They did not touch upon Phillips’s call for “a critical look at how geographers and others have conceptualized, measured and interpreted patterns of segregation.” (Phillips, 2007, p. 1139). In another review focusing on spatializing segregation measures (Wong, 2016), I questioned the validity of existing conceptual foundations of segregation measurement, which has been dominated by the three-decade-old segregation dimensions proposed by Massey and Denton (1988). While we have been trying to develop more effective measures to differentiate the spatial arrangements of different population groups, the meanings of segregation need to be interrogated further. What does the term “segregation” really mean despite being used in various contexts? Among all the dimensions, clustering seems to be closest to people’s perception of segregation. A concept related to clustering is spatial autocorrelation (SA) and segregation studies have employed SA measures (e.g., Brown & Chung, 2006). Do all these measures, spatial and aspatial, based on multiple concepts really reflect segregation or something else? Segregation is often regarded as undesirable because when people are separated spatially, they are assumed to be unequal (Goldsmith, 2003). But what if separated but equal? Would it be more effective to focus directly on the unequal outcomes rather than on segregation—a surrogate? We occasionally raised these substantive questions, but we have not made much progress in sorting them out.

On the other hand, the research community gradually recognized the multi-faceted nature of segregation. Measuring segregation in a single geographical or social context such as residential, work or school space only is no longer sufficient. The importance of residential space should not be undermined but nonetheless, it is just one of many sociocultural spaces, and exposure to other population groups beyond the residential space should be accounted for (Wissink et al., 2016; Wong & Shaw, 2011). After 2011, many segregation studies adopted an activity-space approach, considering the mobility patterns of individuals beyond the residential space (e.g., Farber et al., 2015; Kwan, 2013; Wang & Li, 2016). We now have a view of segregation more comprehensive than decades ago, and research is no longer confined to the ecological or areal-based approach. Instead, more studies have relied on individual-level data.

Individual-level data come from at least two major sources of different nature. Due to the release of historical census data, individual census records are available, supporting historical and temporal population and segregation studies at very high spatial resolutions (Logan et al., 2011, 2015; Páez et al., 2014). Another type of individual-level data are mobility data, including geo-referenced social media and mobile phone data (e.g., Blumenstock & Fratamico, 2013; Silm & Ahas, 2014). As these data often include time stamps, the temporal dimension can be ingested into measuring segregation (van Ham & Tammaru, 2016). These individual-level spatiotemporal trajectory data also play a role in stimulating research to address the stubborn geographical scale issue in measuring segregation, part of the MAUP. Measuring segregation also has taken the multi-level modeling approach (Fowler, 2016; Harris, 2017; Jones et al., 2015; Manley et al. 2015), although some general ideas may be dated back to decades ago (Moellering & Tobler, 1972; Wong, 2003a, 2003b). A closely related approach is the variable scale approach, which may be exemplified by the bespoke method supported by relatively fine-scale population data available in certain countries or through estimations (Clark et al., 2015; Östh et al., 2014, 2015). To a large degree, these are sensitivity analyses depicting the changes in segregation level when size of neighborhood changes (Wong, 2005). Recognizing the scale-sensitivity of measuring segregation, coherent frameworks are needed to connect multiple geographical levels.

Adding to these methodological challenges has been the data quality issues. ACS has been the main source of socioeconomic and housing data in the U.S. since 2004. Considering the estimate reliability is a major challenge in spatial analysis when using data from ACS and other population and health surveys, such as the US Census’s Current Population Survey (CPS), the US Center for Disease Control and Prevention’s Behavioral Risk Factor Surveillance System (BRFSS) data and the US National Cancer Institute’s Surveillance, Epidemiology, and End Results (SEER) Program. During the past decade, researchers have made some headway in incorporating estimate error in geovisualizing estimates statistically (Kronenfeld & Wong, 2017), determining class breaks in choropleth maps using simple statistical concepts (Sun et al., 2015, 2017) or sophisticated optimization methods (Koo et al., 2017; Mu & Tong, 2019; Wei & Grubesic, 2017), and measuring spatial autocorrelation (Jung et al., 2019; Koo et al., 2019). Therefore, we currently have some tools to analyze population survey data spatially with more honesty by considering error associated with estimates (Koo et al. 2018).

One development in the past decade is worth mentioning. Before 2010, applying the more sophisticated and spatial segregation measures was challenging as some measures required spatial operations not supported by generic tools (Wong, 2003a, 2003b). After 2010, a series of effort relying on open-sourced tools has improved the situation. Apparicio upgraded his C#-based Segregation Analyzer to the R-based Geo-Segregation Analyzer (Apparicio et al., ). Other R-based tools included the “seg” package developed by Hong et al. (2014), and more recently the ambitious attempt by Tivadar (2019) with OasisR. On the Python track, a segregation module was implemented in PySAL (Cortes et al., 2020). Now, we have no shortage of computational tools for measuring segregation. However, reliability of these tools should be tested thoroughly as in the case of testing spatial statistical tools (Bivand & Wong, 2018).

Applying computational and data mining tools has been a major trend in the past decade, and some segregation studies also have taken on the data mining-analytics approach, letting data to drive the inquiries. It is important to ask if those studies are merely exercises to rehash the knowns or do they really advance our understanding of segregation.

## Footnotes

1 The R25 Training Grant was funded through the Eunice Kennedy Shriver National Institutes of Child Health and Human Development (NICHD 5R-25 HD057002; Principal Investigator: Stephen A. Matthews). The specialist meeting was held in Santa Barbara, California, USA and convened by the Center for Spatial Studies at the University of California, Santa Barbara in conjunction with the Population Research Institute at the Pennsylvania State University. Attendees at the 2011 meeting all provided short statements (see: http://ncgia.ucsb.edu/projects/spatial-demography/participants.php) and a Final Report is available at: http://ncgia.ucsb.edu/projects/spatial-demography/docs/Future-Directions-in-Spatial-Demography-Report.pdf).

2 The workshop series had focused on topics including point pattern analysis, spatial econometrics, geographically weighted regression, and multi-level modeling (Matthews and Parker, 2013).

3 Whereas the majority of attendees were demographers and geographers, many disciplines were represented, including anthropology, economics, epidemiology, health economics, and political science. Most participants were driven by their interests in demographic research questions. The geographical coverage limited to the United States was unfortunate. In this collection of short commentaries, we have tried to rectify the limited geographical coverage though the N of contributors is inevitably small. We have reached out to additional researchers and we hope to include additional commentaries in a future issue of the journal from Central and South America and from Europe.

4 One of the developments of the past decade was the launch of this journal by Frank Howell and Jeremy Porter (Howell and Porter, 2013). Frank was an invited participant at the special meeting, and during the meeting spent time sowing the seeds of the journal and recruiting people to be editorial board members.

5 Deborah Balk presented at the meeting and both Katherine Curtis and David Wong attended. TC Yang and Daniel Parker both attended the specialist meeting too; at the time, both were very earlier career scholars. The other commentators and several co-commentators did not attend. The editor welcomes additional comments related to the theme of “looking back, looking forward” from scholars from across the globe. Please contact Stephen A. Matthews at sxm27@psu.edu.

6 Earlier proposals for critical demography can be found in Horton (1999) and Massey (1999).
